# The blue lizard spandrel and the island syndrome

**DOI:** 10.1186/1471-2148-10-289

**Published:** 2010-09-20

**Authors:** Pasquale Raia, Fabio M Guarino, Mimmo Turano, Gianluca Polese, Daniela Rippa, Francesco Carotenuto, Daria M Monti, Manuela Cardi, Domenico Fulgione

**Affiliations:** 1 Department of Earth Science, University of Naples Federico II, Naples, L.go San Marcellino 10, 80138 Naples, Italy; 2 Department of Structural and Functional Biology, University of Naples Federico II. Via Cinthia MSA, Naples, Italy

## Abstract

**Background:**

Many small vertebrates on islands grow larger, mature later, lay smaller clutches/litters, and are less sexually dimorphic and aggressive than their mainland relatives. This set of observations is referred to as the 'Island Syndrome'. The syndrome is linked to high population density on islands. We predicted that when population density is low and/or fluctuating insular vertebrates may evolve correlated trait shifts running opposite to the Island Syndrome, which we collectively refer to as the 'reversed island syndrome' (RIS) hypothesis. On the proximate level, we hypothesized that RIS is caused by increased activity levels in melanocortin receptors. Melanocortins are postranslational products of the proopiomelanocortin gene, which controls pleiotropically pigmentation, aggressiveness, sexual activity, and food intake in vertebrates.

**Results:**

We tested the RIS hypothesis performing a number of behavioral, genetic, and ontogenetic tests on a blue colored insular variant of the Italian Wall lizard *Podarcis sicula*, living on a small island off the Southern Italian coast. The population density of this blue-colored variant was generally low and highly fluctuating from one year to the next.

In keeping with our predictions, insular lizards were more aggressive and sexually dimorphic than their mainland relatives. Insular males had wide, peramorphic heads. The growth rate of insular females was slower than growth rates of mainland individuals of both sexes, and of insular males. Consequently, size and shape dimorphism are higher on the Island. As predicted, melanocortin receptors were much more active in individuals of the insular population. Insular lizards have a higher food intake rate than mainland individuals, which is consistent with the increased activity of melanocortin receptors. This may be adaptive in an unpredictable environment such as Licosa Island. Insular lizards of both sexes spent less time basking than their mainland relatives. We suspect this is a by-product (spandrel) of the positive selection for increased activity of melanocortins receptors.

**Conclusions:**

We contend that when population density is either low or fluctuating annually as a result of environmental unpredictability, it may be advantageous to individuals to behave more aggressively, to raise their rate of food intake, and allocate more energy into reproduction.

## Background

Populations of organisms confined to islands often evolve extensive morphological and behavioral changes often over a short time [1]. The best known of these changes are gigantism in small vertebrates and dwarfism in larger species, the so-called Island Rule [2]. In addition there are changes in body shape. Insular mammals often have reduced limb lengths and enlarged cheek teeth [3]. Many insular birds evolve flightlessness [4,5], dull plumage coloration [[6-8], but see [9,10]], and larger bills [11,12]. Insular rodents exhibit an "Island Syndrome" [13] involving the co-occurrence of most (and often all) of these traits: reduced aggressiveness, gigantism, reduced litter size, greater life expectancy, and delayed sexual maturity. McNab [14] further noted that organisms affected by the syndrome have a reduced basal metabolic rate, and a propensity to enter torpor, which entails reduced energy expenditure [14]. Adler and Levins [13] linked the emergence of the island syndrome to the reduced interspecific competition and predation pressure that, they claimed, are typical of islands, and to the size and degree of isolation of the island itself. When predation pressure is low insular population size increases rapidly (= density compensation and overcompensation). This makes it adaptive to invest more in somatic growth and less in reproduction, laying smaller litters/clutches of larger offspring [15-19]. In crowded conditions larger adults and larger offspring usually have higher fitness and better survival [20-24].

Since Adler and Levins's seminal work, it has been suggested that a similar "syndrome" occurs in insular passerine birds (reviewed in [25]). According to this theory these birds tend to evolve larger body size, grow larger bills, are less sexually dimorphic and less aggressive towards conspecifics, reach sexual maturity later in life, lay smaller clutches of larger offspring, live at higher density, and have higher habitat fidelity than their mainland relatives. It is apparent that when living at high and stable population density, many insular vertebrates show a number of correlated trait shifts including, at least, decreased sexual dimorphism, decreased energy allocation to reproduction, and decreased aggressiveness toward conspecifics. Here we refer to these trait shifts as the "Island Syndrome" expanding upon the original definition given for rodents [13].

Similar observations have been made for reptiles. For instance, lizards on islands show a host of trait changes including melanism, modified limb lengths and head shapes, size change (i.e. dwarfism or gigantism), smaller clutch size, frequent shifts to herbivorous diets, and reduced aggressiveness [22,26-37]. Buckley and Jetz [38] reported that insular lizards often live at very high densities (ca. 1900 individuals per Ha^-1^) hence density compensation probably takes place in many insular lizard populations.

Under the Island Syndrome fecundity, aggressiveness and sexual dimorphism decrease because the population density is high and stable, and the environment is predictable. Here we hypothesize that when the environment is highly unpredictable and there are strong fluctuations in population size, investment of more resources into reproduction, aggressive behavior, and sexual competition are favored [28]. Sexual dimorphism is expected to increase because of the greater emphasis on sexual selection [24]. The same set of trait shifts should theoretically occur when predators are present and keep the population density low. We name this hypothesis the "Reversed Island Syndrome" (RIS, see Table 1).

**Table 1:** Phenotypic traits shifts expected to occur in insular populations as compared to their mainland relatives according to the Island Syndrome and to the RIS.

trait	Island syndrome	Reversed Island syndrome (RIS)	Trait changes mediated by increased POMC activity (the involved MCR is indicated)
Population density	high	Low or inconstant	-

Aggressiveness	low	high	high, MC5R

Body size	usually large	small in endotherms, either small or large in ectotherms	small, MC3-4Rs

Energy expenditure	low	high	high, MC3,4R

Sexual dimorphism	small	high	-

Development	-	faster, or anticipated	-

Voracity*	low	high	Either low or high, depending on the balance between MC3R and 4R

Pigmentation*	irrelevant	darker, but may occur in non-melanistic species as well	darker, MC1R

Shifts in food intake rate and pigmentation (indicated with an asterisk) are not directly predicted by the RIS hypothesis and are only true if RIS is triggered by changes in melanocortin receptors activity, and these are not polymorphic. In the last column we report the trait shifts mediated by increase in POMC expression and their relationship with RIS.

In mammals, clutch size generally decreases with increasing body size [21], thus gigantism is likely to be associated with lower reproductive effort/fewer offspring. In reptiles, on the other hand, large size is associated with larger clutch and litter sizes [39]. Moreover, large insular reptiles may have simply lived for longer [40]. Consequently, we predict RIS applies may apply to dwarf insular mammals and to large insular reptiles.

We provide explicit tests of the RIS-predicted phenotypic and behavioral trait shifts (Table 1), by using the Licosa Island Wall lizard *Podarcis sicula klemmeri *as a model. This is a large-sized, melanistic variant of the Italian Wall lizard *P. sicula *from mainland Italy [41,42]. *P. s. klemmeri *population size fluctuates yearly by up to 40% (Tables 2 and Additional file 1: Table S11).

**Table 2:** Lizards' density estimates on both the Licosa Island and on the facing mainland strip of Punta Licosa, over a 5-years survey (see Additional file 1: Table S11 for further details).

Estimated population density		
Year	Licosa (island)	Punta Licosa (mainland)
2004	148.1	355.3
2005	246.2	341.1
2006	231.2	360.9
2007	241.7	388.0
2008	159.4	350.9

**Figure 1:**
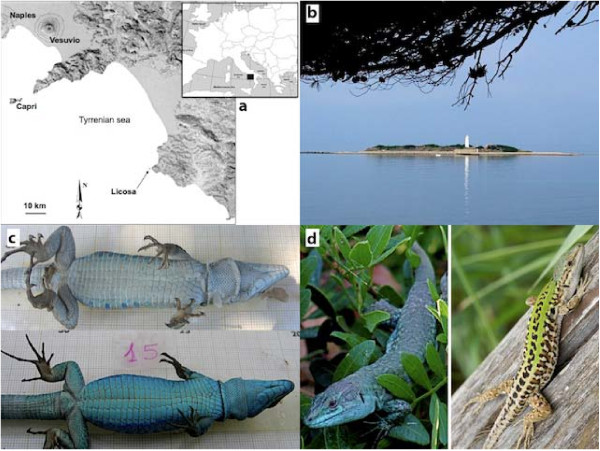
**Study site location and lizards color morphs**. Location (a) and picture (b) of the Licosa Islet taken from a foothill on the facing mainland strip of Punta Licosa. Comparison of island and mainland skin color phenotypes (c and d, blue morphs are insular).

Individuals of both sexes from the insular population are bright blue and no normal colored lizard (with a green back and pale undersides) has ever been observed on Licosa (Figure 1). We predicted that *P. s. klemmeri *would be more aggressive, more sexually dimorphic, and develop faster than mainland *P. sicula*.

We investigated the genetic basis of *P. s. klemmeri *body coloration. The blue color is a form of melanism in reptiles [43]. In vertebrates, melanistic coloration is often associated with increased aggressiveness and sexual activity [44-46]. In a recent review Ducrest et al. [46] suggest the existence of a behavioral syndrome resulting from the pleiotropic effects of the proopiomelanocortin (POMC) gene, the products of which regulate genes for melanocortin receptors, MCRs. POMC produces the melanocortins α-, β- and γ-MSH and ACTH, which bind to five melanocortin receptors (MC1-5R). In vertebrates, POMC and the five MCR genes are highly conserved and their tissue distribution and functions are similar across species [47]. MCRs are involved in the regulation of pigmentation, aggressiveness, food intake, energy expenditure, sexual activity, and immunological responses to stress factors. Since these coordinated traits partly overlap with the trait-shifts we predicted to occur under RIS (Table 1), we predicted that increased MCR expression levels could be the proximal, physiological causes of the RIS. We tested this hypothesis by comparing levels of expression of three different melanocortin receptors: MC1R, 3R, and 4R; which directly influence skin pigmentation (MC1R), food intake rate, body size, and energy expenditure (MC3R and MC4R), and sexual activity (MC4R) (see [46]).

These predictions hold only if there are no mutations in MCR genes [46]. Therefore, we sequenced and compared the MC1R locus in the two populations in order to rule out the possibility that the striking difference in pigmentation in our lizards populations is due to mutation in MC1R in the island population. We predict that activity for all of these MCRs is higher in the insular lizards. Increased aggressiveness and sexual activity are expected to occur if an over-expression of the melanocortin system is the molecular basis of the RIS, whereas changes in food intake rate and darker pigmentation are expected to occur irrespective of RIS, as a consequence of the pleiotropy in the melanocortin system (Table 1).

Finally, we investigated head shape ontogeny in Licosa and mainland *P. sicula *to test for differences in developmental and growth rates. Ontogeny is particularly relevant here because life history theory predicts a tradeoff between growth and reproduction [21]. Changes in ontogeny may be reflected in adult shape differences because individuals that mature earlier may appear paedomorphic [48]. Paedomorphosis is linked to increased reproductive investment in ephemeral habitats in amphibians [49], and was proposed to drive gigantism in Canary Island lizards *Gallotia stehlini *and *G. simonyi *[50] and dwarfism in sauropod dinosaurs [51] and dwarf elephants [52]. On the other hand, positive selection for sexually-selected traits may produce peramorphosis, since sexually selected traits usually appear later in ontogeny [53]. Under RIS, we predicted that the insular lizard (1) develops faster in order to mature early, (2) is more sexually dimorphic as a consequence of strong sexual selection, and (3) has slower growth rate as a consequence of re-allocation of resources from growth into reproduction [21].

## Results

### Behavioral traits shifts

**Figure 2:**
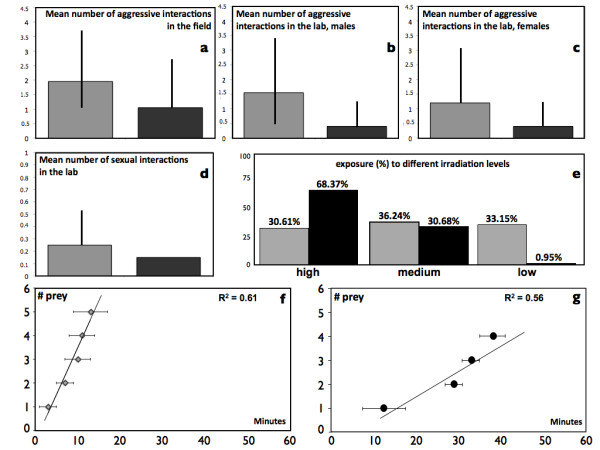
**Behavioral trait differences between mainland and insular lizards**. Mean number of aggressive interactions in the field (a), and in the lab (partitioned per sex, b, c). Mean number of sexual interactions per hour (d) percent differences in exposure to heat between the two populations (e). Mean time to prey consumption is reported in (f) Licosa and (g) Punta Licosa, over one hour of observation. Grey shades refer to the insular populations; black shades refer to mainland populations. Bars in histogram plots indicate 1^st ^and 3^rd ^quartile.

Insular lizards were much more aggressive, spent less time basking and had higher food intake rate (Figure 2). In the field (Figure 2a), we observed island lizards either threatening or attacking other individuals, on average, 1.95 times per hour, compared to 1.05 times on the mainland. A randomization test indicates this difference in attack rate (0.90) is highly significant (random sets, mean difference of the means = 0.385, p = 0.004). In the lab (Figures 2b and 2c), insular males were nearly four times as likely to attack other males as mainland males (randomization test: mean difference = 0.210, p < 0.001). Attacks towards the same sex were significantly more common for insular than for mainland females (1.2 versus 0.4 attacks per hour; randomization test: mean difference = 0.80, p = 0.010).

Sexual interactions in the lab were quite rare (Figure 2d). We counted just three interactions in mainland lizards and five in insular lizards. The difference is not significant (p = 0.208). Plainly, though, the power of the test is very low given the small numbers.

Lab experiments revealed that insular lizards had higher food intake rate than mainland lizards. Mainland lizards ate wax worms, on average, at 7'45" minute intervals, insular lizards at 3' 2" intervals. On average, insular lizards attacked their first prey after 4' 6'' on average (range 3'15'' to 7'01"), whereas mainland lizards did not begin feeding until, on average, 12' 26'' (range 9'34" to 21'00"). All insular lizards consumed all the wax worms they were given within 12 to 20 minutes while none of the mainland lizards consumed all the wax worms, and the 4^th ^wax worm was consumed after 28 to 43 minutes (Figures 2f and 2g). Regressing time (minutes) to prey consumption versus the number of prey consumed gives a slope of 0.39 for insular lizards (n = 20) and 0.11 for mainland lizards (n = 20). The two slopes are statistically different (t = 2.85, p = 0.007).

In the field, the difference between the two population in the time spent basking is not significant (means: mainland 28'22'', island 28'30''; t = -0.040, p = 0.96). Yet, in the lab mainland individuals of both sexes spent about twice as much time close to a heat source (a light bulb) than insular ones (Figure 2e). Insular lizards spent about equal amounts of time under different levels of radiation while mainland lizards spent 68% of the time directly under the light bulb. The difference between the two populations is highly significant (chi-squared = 1104, df = 2, p < 0.001).

### MC1R gene polymorphism and selection test

Unlike all other vertebrates studied, we discovered that the MC1R gene in *P. sicula *consists of two exones. The first codes for the entire protein (314 aa), and the second includes the stop codon and 3' UTR (see Additional file 1, the sequences were deposited in Genbank under the accession number GU225767).

Three nucleotide polymorphic sites occur in the two populations. One of them (T-110-I) results in a charge-changing amino acid variant in the first extracellular loop of MC1R. The extracellular regions of the MC1R-encoded receptor are important for ligand binding and regulation of MC1R reactivity. This amino acid variant creates two different alleles, but we did not find statistical association between the T-110-I polymorphism and pigmentation (Fisher's Exact Test, p = 0.23, n_island _= 20, n_mainland _= 20). Thus, the absence of association between MC1R polymorphism and skin color rules out the possibility that mutations in MC1R caused the differences in pigmentation we observed between our study populations.

Fifty-five percent of the sequences (= 11) were associated with isoleucine at amino acid site 110 in the Licosa island blue lizard, whereas 45% of sequences were associated with threonine at this position. The corresponding figures in the mainland population were 25% and 75%, respectively (see Additional file 1: Table S12).

Patterns of nucleotide variation within lizard populations were consistent with a neutral model of molecular evolution: the distribution of allele frequencies at MC1R as reflected in Tajima's D is generally consistent with neutral expectations for mainland and island populations. For the island population we found Tajima D = 0.70, number of segregating sites S = 2, nucleotide diversity π = 0.00082; for mainland Tajima D = 1.05, number of segregating sites S = 2, nucleotide diversity π = 0.00091.

### Differences in MCRs expression

**Figure 3:**
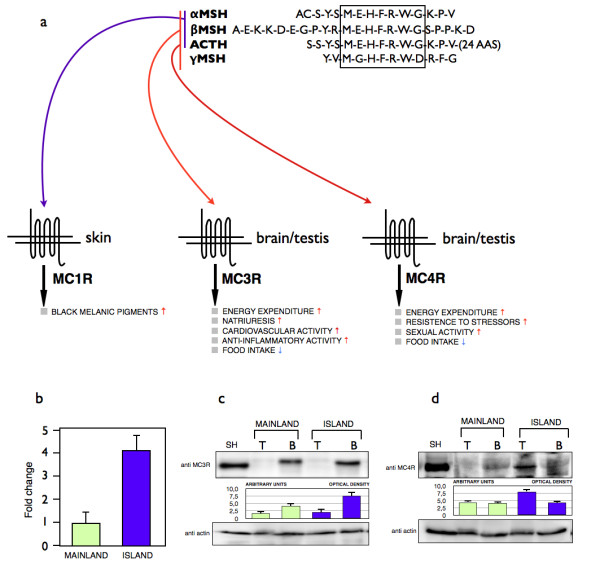
**The melanocortin system**. Top, the proopiomelanocortin (POMC) gene produces the melanocortins (α-, β- and γ-MSH and ACTH) which bind to melanocortin receptors. Amino acid sequences of the melanocortin peptide and core heptapeptide aminoacids are highlighted (a). The tissues were we have localized three MCRs receptors are shown, along with the functions they control; upward arrow means "increased function". Total RNA screened for MC1R mRNA transcripts by real-time RT-PCR, much increased expression of MC1R transcripts is evident in the skin of island lizards (b). Brain and testis expression of the melanocortins receptors as evidenced by western blotting with anti-MC3R and anti-MC4R antibodies: c and d respectively. T, lizard testis; B, lizard brain; SH, human neuronal cells. Actin and SH were used as controls.

MC1R expression varied between the populations with the insular population showing a roughly four-fold greater gene expression compared to the mainland population (Figure 3a) (t-test_one-tailed_, t = 3.51, p = 0.0012, df = 19), suggesting that *Podarcis *MC1R gene expression could be involved in skin pigmentation.

Western blot analyses showed significant differences in the expression of MC3R and MC4R in the lizards' brain and testes, using actin as an internal standard (Figures 3b and 3c). MC3R protein expression in the brain of insular lizards was about three times higher than in mainland lizards' brains (range: 1.5-5.3, n = 5; t-test: t = 4.506, p = 0.001). No appreciable difference in MC3R density was noted in male testes between the two populations (t-test: t = 0.355, p = 0.730).

We found that MC4R expression in testes of insular male lizards was almost twice that observed in mainland lizards (range: 1.4-3, t-test: t = 2.445, p = 0.037). Brain expression of MC4R was not significantly different between the two populations (Figure. 3c, t-test: t = 0.402, p = 0.697).

### Growth and development

#### Age, size, and growth

Insular lizards had larger maximum SVL (island SVL_mean _= 71.84, n = 80; mainland SVL_mean _= 65.54, n = 92; t = 2.644, p = 0.009). All three lizards > 80 mm SVL belonged to the insular population, and they were also the only lizards in our sample bearing 4 lines of arrested growth (LAGs, we assumed 1 LAG = 1 year of age) in their long bones. Island lizards were a little older, on average (Licosa: mean number of LAGs = 2.21; median = 3; Punta Licosa mean number of LAGs = 1.52, median = 1, Wilkoxon test, p = 0.001). In addition, the sex ratio was different, being nearly 1:1 on the mainland, but about 1:2 (males:females) on the island (Punta Licosa 47M-45F; Licosa 31M-59F, Fisher exact test: p = 0.04). We are unaware of a sampling bias that might have increased the likelihood of capturing females on the island.

Growth rates did not differ between the two populations (Table 3). When sexes were analyzed separately, however, females grew significantly slower on the island. In fact, insular males grew faster than insular females. This accounts for the significant body size dimorphism on the island (Table 3).

**Table 3:** Growth rates (in mg * day-1). Lic = Licosa (island); Plic = Punta Licosa (mainland).

	Growth Rate	95% CI	n
Lic Female	238	224-255	36

Lic Male	316	291-351	25

Plic Female	291	255-342	37

Plic Male	303	269-348	43

CI = confidence intervals.

#### Changes in ontogeny

A standardized major axis regression of procrustes distances calculated from a common reference (the consensus shape of the five shortest individuals) indicated that the slope (rate) of somatic development did not differ between populations [Licosa Island: n = 61, slope = 0.0183 (95% CI: 0.0147-0.0220), R^2 ^= 0.560, p < 0.001; Punta Licosa: n = 80, slope = 0.0222 (95% CI = 0.0182-0.0272), R^2 ^= 0.465, p < 0.001; test for common slope: p = 0.205].

The multivariate angle between regression vectors (of shape versus age) of the two populations was 59.8, the angle within Licosa Island population was 14.8, and within Punta Licosa was 64.6. This means that although the between-populations angle was rather large, the ontogenetic trajectories of the two populations were the same (see Additional file 1: Figure S6).

Procrustes distances between age groups confirm this notion (Table 4). The distances between juvenile individuals of the two populations, and between adults of the two populations are similar, implying the ontogenetic trajectories are parallel (Table 4). Importantly, the distance between the two groups of juveniles is significantly > 0, indicating that consistent difference in development between the two populations occurs early in morphogenesis [100]. The largest distance (0.067) was between Licosa adults and Punta Licosa juveniles. This distance was much larger than between Licosa juveniles and Punta Licosa adults (Table 4). Finally, the lengths of ontogenetic vectors were 0.057 and 0.055 on Licosa and Punta Licosa, respectively.

**Table 4:** Shape distances between groups of individuals per population.

		Island		mainland	
		**juveniles**	**adults**	**juveniles**	**adults**

island	j	0	< 0.001	< 0.001	< 0.001
	
	a	0.057 (0.051-0.068)	0	< 0.001	< 0.001

mainland	j	0.034 (0.028-0.050)	0.067 (0.055-0.083)	0	< 0.001
	
	a	0.046 (0.039-0.058)	0.035 (0.031-0.045)	0.055 (0.046-0.067)	0

The metric of shape distance is the Procrustes distance (see text for explanation). Juveniles are assumed to be all the individuals of age 0-1 LAGs. Individuals with n > 1 LAG are considered adults. P values are reported in the upper-right triangle, Procrutes distances (with 95% confidence intervals) are in the lower triangle. j = juveniles; a = adults.

Taken together, these results indicate that although the rate and trajectory of somatic development is similar between populations, insular juveniles head shapes appear more adult-like, and insular adult head shapes develop "beyond" mainland adults, in keeping with the notion of peramorphosis. The shift occurs early in morphogenesis (see Additional file 1: Figures S6-9).

### Sexual dimorphism

In two-factor ANOVA, residuals of OLS regression of log SVL against log head width revealed significant differences between populations (F = 7.097, p < 0.001, df = 1) and sexes (F = 28.382, p < 0.001, df = 1). The interaction term is significant (F = 13.258, p < 0.001, df = 1). Insular male heads are much larger than insular female heads. Insular male lizard heads are also larger than mainland lizard heads in both sexes (see Additional file 1: Figure S5 for further information).

Sexual size dimorphism (SSD) is not particularly pronounced in *Podarcis *lizards [54]. We found significant SSD only in three year old insular lizards. Males' mean SVL at three years was 78.3 mm on Licosa, whereas females' mean SVL was 69.3 mm. The SVL difference was statistically significant (p < 0.001; see Table 5 for comparisons of all age groups).

**Table 5:** Sexual shape and size (SSD) dimorphisms.

sexual shape dimorphism				
	**island**	**p**	**mainland**	**p**

*d*	0.0382 (0.0347-0.0504)	< 0.001	0.0309 (0.0278-0.0513)	0.05

				

sexual size dimorphism	Mean SVL: Females/Males (# of individuals)			

Years (LAGs)	island		mainland	

1	42.02(6)/40.19(5)		47.40(14)/45.36(7)	

2	57.85(6)/62.02(5)		59.95(9)/59.85(13)	

3	69.71(11)/78.28(8)*		72.55(7)/76.90(6)	

*d *= Procrustes distances. Number of individuals is reported in parentheses. For SSD we report the mean SVL of females (left) and the mean SVL of males (right). *t = 4.26, p < 0.001.

Sexual head shape dimorphism was large. The procrustes (shape) distance between insular adult male and female individuals was 0.382 (p << 0.001, Table 5). On the mainland, this distance was smaller and only marginally significant (0.302, p = 0.05, Table 5).

## Discussion

Despite the fact that the two populations analyzed here diverged a short time ago, and that there is sizeable genetic introgression between them [42], we found striking evolutionary change in the Licosa lizards. Compared to their mainland relatives, these blue lizards were much more aggressive, voracious and dimorphic, as predicted by the RIS hypothesis (Table 1). At first glance the increased activity in both MC3R and 4R is expected to decrease food intake. Yet, MC3R is believed to function as an inhibitory autoreceptor on POMC neurons: experimental injections of MC3R-specific agonists stimulate feeding in mice [55]. Moreover, food deprivation increases MC4R expression in the liver of barfin flounder *Verasper moseri *[56]. In our insular lizards, the complex pathways that regulate food intake via modification of MC3R and 4R activity deserve further investigation. However this is beyond the scope of the present study.

The Island syndrome predicts that sexual selection should be less intense in insular vertebrates living at high and stable population density, because mates are a non-limited resource in this condition [24]. Conversely, with either low or strongly fluctuating population size, as with the RIS hypothesis, higher investment in (early) reproduction should favor positive selection of sexually-selected traits, due to an unpredictable mortality schedule [21,24]. In keeping with this, insular males had larger heads, and sexual size and shape dimorphism were increased on the island (Table 4, Additional file 1: Figures S5, S7, and S8). Moreover, in insular females body size growth rate was low (Table 3), which is expected when energy allocation toward reproduction is increased. As predicted, somatic development proceeded at a faster rate early in insular lizards' ontogeny, as indicated by head morphogenesis (Additional file 1: Figure S9).

The notion that the Licosa lizard is a large-sized variant of mainland *Podarcis sicula *[41,42] was confirmed by our data. However, insular lizards were, on average, older, and the higher mean SVL on the island could be a result of different age structure (perhaps related to lower predation) rather than the result of direct selection on size.

The increased density of MCR receptors in insular lizard tissues provides a proximate explanation for the phenotypic trait shifts we observed in these organisms (Figure 3). It is remarkable that we found neutral selection on the MC1R gene both in insular and mainland individuals. This suggests that selection for color per se probably does not occur. Although we found some evidence that the darker coloration of insular lizards decrease the time spent basking the blue color may simply be a by-product of concerted increase in MCR activity levels. Consequently, we argue the blue coloration may have been recruited adaptively for better thermoregulatory activity, as a 'spandrel' [57].

We argue that increased aggressiveness and food intake rates are the likely targets of natural selection in our insular lizards. Aggressive individuals are very often the most successful (in terms of fitness) in natural populations, although they incur a greater risk of injury and greater exposure to predators. However, the risk of becoming prey is very low on Licosa. Behaving aggressively and increasing food intake rate should be very advantageous in a restricted habitat with limited and probably inconstant access to both resources and mates, as is the case with Licosa.

In lizards, color morphs are quite common [58-60] and often underlie differences in life history traits and reproductive strategies even in the same population [60-67]. In insular lizards, black and blue morphs are frequent (e.g. the Sombrero black lizard *Ameiva corvina*, the Brusnik black lizard *Podarcis melisellensis pomoensis*, dark *Podarcis lilfordi *on Isla del Rey, and a number of blue-colored *Podarcis *distributed in many Mediterranean islands), and evolutionary mechanisms similar to what we describe for *P. s. klemmeri *may have been working in many of them. Bauwens and Castilla [68] noted that all the largest *Podarcis lilfordi *males on Cabrera island have black or deep blue skin, and indicated that this change in coloration is ontogenetic, occurring at SVL of 64 mm. Furthermore, they noted that the largest adults of all of the 18 islets surrounding Cabrera are black.

Males of Bonaire island whiptail *Cnemidophorus murinus *occur in two color morphs, brown and blue. Baird et al. [69] found that blue males are far more aggressive than browns. Their sexual activity is much increased, and their testes are larger. Of all the courtship encounters they observed 85.7% were initiated by blue males, vs. 7.1% initiated by brown males and 7.2% initiated by females. Individuals of a translocated population of St. Lucia whiptail *Cnemidophorus vanzoi *on Praslin Island [70] evolved increased growth rates, and high intensity of intraspecific competition (see also [37,71,72] on Balearic *Podarcis lilfordi *and on Skyros archipelago *Podarcis gaigeae*). Praslin whiptails also have increased sexual size dimorphism and sexual dichromatism. Moreover, very surprisingly, Praslin whiptails are territorial although *Cnemidophorus *belongs to a family (Teiidae) of typically non-territorial lizards [70]. Some adult males also maintain juvenile colors [70].

In practice, these studies suggest that the occurrence of the 'reversed island syndrome' in insular lizards may well extend to other melanistic forms. However, we emphasize, that the link between RIS and melanism is not necessary (see Table 1). The mechanism behind the emergence of RIS is the reallocation of energy from growth to reproduction. This reallocation is expected to take place when resource supply and/or mortality risk are unpredictable, thereby favoring aggressiveness and voracity to secure resources and mates in the short term [21].

For example, the Okada broad-headed skink *Plestiodon okadae *occurs in a number of islands of the Izu Archipelago, Japan. Hasegawa [28,73,74] noted that some *P. okadae *populations have: "a suite of traits including small body size, early maturity, production of small and many eggs, low frequency of failure in follicle development, annual reproduction, high relative clutch mass and low post-reproductive mass of females. Whereas others have a suite of the opposite character states". Fast life histories occur on the island with the largest predator fauna (Ohshima), whereas slow life histories characterize islands with higher density and the lowest mortality of hatchlings (Miyakejima and Aogashima). Still, fast-living Ohshima skinks are the smallest and the most dimorphic, with males having disproportionately large heads with a distinctive color pattern [73].

Hasegawa manipulated lizard density in Miyakejima [74], removing adults in order to simulate increased mortality. As expected, after the experiment, both males and females started reproducing at a younger age, and females laid larger clutches, whereas egg size and body size at first reproduction went almost unaltered. This experiment demonstrates how decreasing survival rates ignited RIS-like changes in life history traits. *Plestiodon okadae *from different islands may well represent both the Island Syndrome and RIS. A similar pattern of variation has been shown for Canary Islands *Gallotia *[75-78].

Island Syndrome and RIS describe a number of phenotypic traits that often, but by no means always, change together in insular species. We think one should not expect that all these traits co-occur. For instance, the most obvious trait shift linked to the Island Syndrome (and we argue, to RIS as well) is change in body size. Taking into account possible changes in ontogeny (in the insular species), the relationship between body size and the Island Syndrome may in fact appear weak. Ontogenetic shifts may produce early-maturing, fast-growing small individuals via paedomorphosis. This is suggested to occur in the extinct dwarf elephant *Elephas falconeri *[52] and in dwarf dinosaurs (e.g. *Europasaurus holgeri *[79]). Slow growth was found to occur in New Zealand's gigantic moas [80,81], extinct giant lemurs of Madagascar [82], females of the Licosa Island lizard (this study), and extinct dwarf Balearic goats [[83], but see ref. [84]]. Bunce et al. [85], however, showed that *Dinornis *Moas were exceptionally dimorphic, and suggested that these birds invested a great deal of energy into egg production. This goes against the Island Syndrome predictions, despite *Dinornis *being huge even by moa standards.

Lancaster et al. [86] correctly noted that resource allocation trade-offs are best understood at multiple levels, and pointed out that "the more resources are available, the less trade-offs will be observed on any level. This occurs over an individual's lifespan for plastic resource allocation traits (e.g. [87]) and over evolutionary time for those mediated by antagonistic pleiotropy or other genetic mechanisms". This means that looking at one aspect alone (e.g. body size, population density, clutch size, etc.) when comparing an insular species to its mainland relative may be misleading.

Spencer et al. [88] emphasized that although growth is expected to be slow when population density is high, rapid growth beyond a critical size reduces predation risk and could be favored (see [22] on *Sauromalus*). Roth [89,90] argued that density compensation might explain dwarfism in insular pigmy elephants. Bonaire whiptails live at huge population density, yet the blue morph males allocate a great deal of resources to reproduction, which is consistent with RIS hypothesis [69]. Bonaire whiptails are herbivorous, and so food is not a limiting factor on Bonaire [91]. We remark once more that it is not density (or body size) per se, but the set of ecological conditions favoring either small or large allocation of available resources toward reproduction that produces the Island Syndrome or the RIS, respectively.

RIS is most likely to occur on small islets where environmental variation (in terms of resource supply and the impact of natural accidents) from one year to the next can be high. The possible instances of RIS described above all relate to species living alone or almost alone on islets and cliffs such as Licosa, Brusnik, Praslin, Isla de la Reya, the islets off the main island in the Cabrera Archipelago, Sombrero, the Faraglioni cliffs off the island of Capri and Vetara islet (the two latter cases are also blue-colored *Podarcis sicula *off the Western coast of Southern Italy).

We showed that the RIS is linked to increased activity of melanocortin receptors in our insular study population. Melanocortins pleiotropically regulate a number of processes including pigmentation, sexual activity, aggressiveness, bodily growth, and food intake. We find it highly likely that the same pattern of change in MCR activity occurs in other melanistic insular organisms.

We believe that shifts in growth rate, body size, age and size at maturity, and clutch size will depend on how these traits trade off with each other [86]. Furthermore, an insular species may well express the RIS without being melanistic.

Despite all of the evidence we present in favor of RIS, there are drawbacks to two-taxon comparative studies [92]. In these studies, it is possible to interpret as adaptation what really is the phenotypic difference between taxa due to other factors or even to genetic drift. In this study, we did not demonstrate that RIS applies to other insular populations/species. However, blue *Podarcis sicula *are common on other islands, and it would be very surprising if the basis for this coloration changed from one island to another. Roulin and Salamin [93] recently demonstrated that insular barn owls *Tyto alba *of the world have lighter pheomelanic coloration and are less sexually dimorphic than continental barn owls. They interpreted this observation as a component of the Island Syndrome, mediated by changes in melanocortin expression. This suggests that, exactly as we predict in our study, POMC involvement in the Island Syndrome and its opposite, the RIS, is common in insular vertebrates.

## Conclusions

We predicted that insular vertebrates facing unpredictable resource supply and mortality schedules should evolve aggressive behavior, high food intake rate, and increased energy allocation to reproduction as a mean to increase fitness. We refer to these predictions as the Reversed Island Syndrome (RIS) hypothesis, in contrast to the Island Syndrome [13]. On the proximate level, we demonstrated that the increased activity of melanocortin receptors underlies RIS in our study organism, the insular wall lizard *Podarcis sicula klemmeri*.

Although much study is needed to fully understand patterns of trait evolution and covariation in insular vertebrates, we hope to have shed some light on this complex issue, by showing how disparate factors must be taken into account in order to gain a meaningful depiction of the evolution of life on islands [94].

## Methods

### Study site and organism

The study population of the Italian Wall Lizard *Podarcis sicula klemmeri *is confined to the small islet of Licosa (ca. 0.8 ha in surface area, geographical coordinates: 40°15'04.23"N, 14°54'01.64"E) [95]. This islet is 400 meters away from the mainland. The island's vegetation is dominated by *Pistacia lentiscus*. Vegetation is nearly absent along the coast.

The Licosa lizard population lives at low (for an island) and fluctuating density. We estimated a maximum population size of only 250 individuals (see Additional file 1: Table S11), that is lizard density is some 300 lizards per Ha (one-sixth of the average for lizard in islands around the world [38]). In five years of field censuses (2001-2006) we noted the number of individuals may change by up to 40% from one year to the next (range: 148.1-246.7, mean: 205.3, see also [42]). Since no freshwater source is present on the islet, mortality can be intense in drought years. Summer drought in one year is significantly correlated to population crash in the next year on Licosa, but not on the mainland (Additional file 1: Table S11).

There are no resident predatory mammals, reptiles or birds on Licosa. Gulls (*Larus michahellis *and *Larus ridibundus*) may be present, principally during the winter. We noted that lizards supplement their diet with gulls' regurgitates even in the presence of gulls. Individuals of migratory herons (*Ardea purpurea*, *Ardea cinerea *and *Egretta garzetta*) occasionally visit the islet during late summer, before their winter migration. Kestrels, *Falco tinnunculus*, were very rarely observed on Licosa during our field surveys.

These observations suggest that although some predation on Licosa lizards is possible, it was minimal during our 5-year census. In contrast, mainland lizards are intensively preyed upon by kestrels, grass snakes (*Natrix natrix*), western whip snakes (*Hierophis viridiflavus*), and feral cats.

*Podarcis sicula klemmeri *is bright blue (Figure 1c and 1d). Melanism is common in Mediterranean island lizards [95]. Our mainland populations, just opposite the island on Punta Licosa (40°15'06.15"N 14°54'19.68"E) are invariably normally-colored with the usual green back with white undersides and little blue dots running along the trunk sides that appear brighter during the breeding season. We sampled lizards from Punta Licosa as the reference (mainland) population to compare with the insular population (Figure 1). All experiments described below were performed in accordance with local and national guidelines governing animal experiments (86/609/CEE and its modifications).

### Behavioral shifts in *Podarcis sicula klemmeri*

We predicted that Licosa island lizards should be more aggressive, sexually dimorphic, and voracious than their mainland counterparts. Change in voracity (food intake rate) is not directly predicted by RIS, but we expected it to occur if increased MCR expression triggers RIS, as we hypothesize.

To test these predictions we conducted both field observations and lab experiments. Field observations were performed during the warmer months, from February until November, when *Podarcis sicula *lizards are active in Southern Italy [96]. During 2006 we performed two sets of observations, in the field and in the laboratory, under controlled conditions. In the field, observations were arranged as follows: we observed, for 1 hour, all individuals from a fixed position, surveying some 10 square meters, in 40 different and non adjacent plots chosen as to maximize inter-plot distance in order to reduce the potential for pseudoreplication. Each plot was visited once. A total of 5 days of observations were performed over a 9 month period. During each observation trial we counted i) instances of intraspecific aggression, including both fights and aggressive displays, and ii) breeding attempts and copulations.

In the field we recorded the total time individuals spent either in full sun or in the shade. Observations of individual lizards were included in the analyses if they lasted for no less than 20 minutes (> 40 hours of observations in total). The mean percentage of time lizards spent in the shade or in the sun, for each observation, was our index of exposure to heat.

Our field observations may be severely affected by incomplete data due to the difficulty of monitoring individual lizards over a sufficiently long time span. We therefore conducted a lab experiment in terraria (measuring 1000 × 1000 × 500 mm) under controlled conditions. Forty adult individuals per population (20 males and 20 females) were kept in laboratory at 25°C temperature. To avoid pseudoreplication, these individuals were collected before field observations began.

We artificially created habitat decorations including wood branches and rocks at the center of the terraria, to allow the lizards either to move close to a heat source or to hide under the rock shelters. The heat source was a 150 watt light bulb hanging 5 cm from the roof of the terrarium and above the branches. We considered three ordinal heating regimes: "low", when the lizard hid under the rocks; "medium", when the lizard stood on the ground exposed to radiation; and "high", when the lizard perched on the branch, close to the heat source. Individual lizards were observed for one hour: we recorded the time spent in each distance class. Then, we computed the average percentage of time spent at each irradiation level and performed a chi-square test to see if the two populations differed.

The experiments on intraspecific interactions and exposure to heat were conducted in June 2006 for both populations. The experiments started 15 days later for Punta Licosa (mainland) lizards because mating occurs 2 weeks later on Punta Licosa than on the island. Both experiments were repeated in June 2007 on a smaller sample to test if differences in population density over years affect the results. Because no significant difference was detected, only the results of the 2006 experiments are reported here.

To study food intake rate in the lab we placed twenty captive individuals (10 males and 10 females) from each population alone in terraria (measuring 500 × 500 × 500 mm) and fed them ad libitum for 5 days. After this period, these individuals were held for 5 days on empty stomach and then presented with 5 prey items simultaneously (caterpillars of Honeycomb Moth *Galleria mellonella*). We video recorded each trial for one hour (the experimenter left the room immediately after the beginning of video recording). We recorded the time elapsed from the beginning of the experiment to the moment each prey item was consumed).

To study intrasexual behavior we held, 10 individuals of each population together in two separate terrariums (5 individuals per 1000 × 1000 × 500 mm terrarium, either all males or all females) fed ad libitum, and video recorded them (in the absence of the experimenter) for one hour them between h8.00 and h18.00 each day, for 20 consecutive days. From each recording session we retrieved the number of intrasexual contests. Threatening displays of the head (e.g. head bobs) were considered signs of aggressiveness toward conspecifics, as were full contests that escalated to fights.

Finally, we studied intersexual activity in two additional terraria (one for each population), by placing 10 individuals per population (5 males and 5 females) together. Individuals in these terraria were video-recorded and observed for one-hour as described above. Instances of intersexual interactions were then counted by examining the films. Chasing of females by males and mating attempts were considered intersexual interactions (regardless of whether they ended up in copulation). Total number of interactions per population and per sex was then computed.

To test for differences between populations in intraspecific activities we performed a randomization test, shuffling data between populations. For number of sexual interactions and aggressive interactions: i) the data were portioned by sex, ii) the number of interactions per individual was shuffled (without replacement) 1000 times at random between populations, iii) for each random set the difference in mean number of interactions per population was computed and iv) real mean difference was compared to the random distribution. Randomization tests were performed both for field and lab observations, although in the field the sex of individual lizards was not discernible by eye; hence field data on aggressive interactions were not partitioned per sex. The use of the randomization test is advisable here since the low number of observations per experiment (see below) makes parametric tests inappropriate.

To test for difference in voracity we first computed the least squares regressions of the (consumed) prey number against the total time since the beginning of the experiment for insular and mainland populations. We then tested for differences in slope between the two regressions lines using a t-test.

All lizard individuals tested in the lab belong to the same size class: 55-65 mm in SVL for females and 60-70 mm SVL for males. These size classes include most adult individuals. A few very large individuals and juveniles were excluded to avoid possible biases introduced by age- and size-specific behavioral and/or physiological differences.

### MC1R polymorphism

We examined the contribution of the melanocortin-1 receptor gene (MC1R) to the skin color pattern in *P. sicula*. In the literature, all known mutations in the MC1R locus, many of which are adaptive, occur in the coding region, either as amino-acid changes or small deletions [97]. However, it is important to note that little is known about the regulatory mechanisms that govern the expression of MC1R at the intracellular level.

The complete MC1R locus was sequenced in seven phylogenetically diverse squamate species with melanistic or normally-colored forms finding that patterns of amino acid substitution across different regions of the receptor are similar, suggesting a conserved function for MC1R in reptiles [97]. Here we cloned the *Podarcis sicula *MC1R gene. We amplified its most conserved region developing the primers by alignment analysis of *Homo sapiens *(AF514787) and *Sceloporus undulatus *(AY586162) sequences. The entire sequence of the gene was then obtained by 3' and 5' RACE experiments. Rapid Amplification of cDNA Ends (RACE) is a procedure for amplification of nucleic acid sequences from a messenger RNA template between a defined internal site and unknown sequences at either the 3' or the 5' -end of the mRNA.

We amplified and sequenced the codogenic MC1R from 20 individuals of each color morph in each population to look for associations between genotype and color phenotype. In a previous study we demonstrated selection was operating on a mtDNA gene sequence of Licosa lizard [42].

We performed Tajima tests for selection on the coding region of the nuclear gene for MC1R. Tajima's D is the difference between two estimates of the expected amount of genetic variation per nucleotide, based on the number of segregating sites *S*, and on average number of pairwise differences ∏, respectively [98]. Under neutral selection, the two estimates of θ should be the same. Therefore, the expected value of Tajima's D for both populations conforming to a standard neutral model of selection is zero. Significant deviations from zero indicate a skew in the allele frequency distribution relative to neutral expectations. Positive values of Tajima's D occur with an excess of intermediate frequency alleles and can result from population bottlenecks, and/or balancing selection. Negative values of Tajima's D indicate an excess of low frequency alleles and can result from population expansions or directional selection. We estimated Tajima's D using DNASP [99].

One way to determine the effect of genetic variation on pigmentation is to analyze the genetic polymorphism affecting receptor function. There are also many complex regulatory mechanisms that govern the expression of MC1R at the intra-cellular level, from gene transcription in response to an external stimulus to the expression of the mature receptor on the melanocyte surface. To examine whether differences in expression level of MC1R influence color patterning, we conducted gene expression assays (reverse transcriptase PCR [RT-PCR] and quantitative-PCR [q-PCR]) on adult skin samples taken from abdominal and neck areas. Specifically, we performed parallel expression analyses for MC1R mRNAs in the two lizard population (n = 5 males and 5 females for each population, see Additional files for full details).

### Western blotting

We tested differential expression of MC3R and MC4R between island and mainland populations by western blotting of testis and brain lysates (n = 10 males per population) and used lysates from human neuroblastoma cells (SH-SY5Y) as a control. Protein concentrations were determined chromometrically with the Bradford assay (Sigma). Samples of equal amounts of protein (100 μg) were subject to SDS/PAGE and then transferred onto PVDF membranes and tested with the appropriate antibody. Mouse polyclonal antibodies anti-MC3R (Sigma™) and human polyclonal antibodies anti-MC4R (Santa Cruz) were used at a 1/2,000 dilution. Rabbit anti-actin polyclonal antibody (Sigma) was used at a 1/1,000 dilution. Secondary antibodies were from Pierce and used at a 1/20,000 dilution. Detection was performed with the peroxidase-based Super Signal West-Pico procedure (Pierce) according to the manufacturer's instructions.

### Growth and development

#### Age determination

Age data are of fundamental importance for the study of ontogenetic trajectories, because the correlation between size and age is rarely linear [21,100]. We obtained age data by applying skeletochronologic standard protocols for other reptiles [101,102]. Skeletochronology was performed both on phalanges and femora of euthanized animals. Bones were decalcified with 3% nitric acid for a time ranging between 1h30' and 3 h depending on their size, rinsed in tap water and cross sectioned using a cryostat. Diaphyseal cross sections (12 μm thick) were stained with Ehrlich's haematoxylin (20') and mounted in aqueous resin. Periosteal lines of arrested growth (LAGs) were then counted by using a light microscope equipped with an image analyzer. As with other reptiles living in temperate regions, we assumed that each LAG corresponds to an annual arrest of individual growth. As a consequence, a lizard's age in years equals the number of visible LAGs. We caution, though, that it is possible to underestimate LAG number because of endosteal resorption (see Additional file 1: Figure S1).

#### Growth rate

Growth rate (in mg/day) was estimated for each sex and population using least squares regression of body weights of individuals versus the number of LAGs transformed into days by assuming each LAG represents a 365 day interval. Individuals with shed tails and gravid or suspected-to-be gravid females (only for the body weight-based growth rate calculation), and individuals in evidently poor conditions (either badly wounded or loaded with parasites) were excluded from the analysis.

#### Geometric morphometrics

Head pictures of sacrificed specimens of known age (= number of LAGs) from post-hatchlings to full adults were analyzed by means of geometric morphometrics. Fifteen landmarks were placed on all head pictures at scale joints, in keeping with the protocol used in Kaliontzopoulou et al. [54] (see Additional file 1: Figure S2 and Table S3). We did not use bilateral landmarks to avoid inclusion of shape variability due to asymmetry. We assessed measurement error by replicating digitations of landmarks on a single specimen ten times. Then, we performed single group analysis of variance with 100 bootstraps. This way we calculated the proportion of variance contributed by measurement error to the variance obtained by using all individuals. This test indicates that only 5.56% of total variance may be due to measurement error (95% CI 4.02-6.91).

Landmark configurations for all individuals were rotated, translated and scaled to unit size via the Generalized Procrustes Analysis (GPA, [103]). Residual shape differences (partial warps) were analyzed via relative warp analysis and thin plate spline (TPS) visualization. Landmark digitation was carried out with the software tpsDig2 [104]. GPA was performed with the software tpsRelw [105]. All the remaining shape analyses were performed with the IMP software suite [106].

The shape data matrix (partial warp scores plus uniform components) of each population was regressed against age (number of LAGs) of each individual to assess head shape ontogeny, taking a single common reference configuration for both populations (the consensus shape between the two smallest individual per population, which were all hatchlings) and then calculating the procrustes distance (the square root of the sum of squared differences in the positions of the landmarks) between each individual and the reference. The two resulting ontogenetic shape vectors (whose length represents the total amount of shape change through ontogeny [100]) were then compared to each other by testing for differences in slopes. The slope represents the rate of somatic development. Data were fitted by reduced major axis regression in the Smatr software [107]. The angle between the two ontogenetic shape vectors (the dot product the regression coefficients of each partial warp component on age) represents the between-vector correlation. When the angle is 0° the vector correlation between them is 1, when the angle is 90° the correlation between them is 0. To test for the statistical significance of the angle, shape residuals were resampled (by means of bootstrap) 999 times within each population to generate 95% confidence intervals for each population (see [98] for details). If the between-populations angle is smaller than the within population range the two ontogenetic trajectories do not differ in direction, but might still differ in length (a case for heterochrony). Otherwise, the two ontogenies are not parallel (a case for ontogenetic allometry, see [100]).

We inspected the length of the ontogenetic vector to quantify the extent of shape change during ontogeny. If this length is shorter in insular lizards and the two ontogenies do not differ in direction (angle) we conclude that insular lizards are paedomorphic. Otherwise, a longer ontogenetic vector would indicate adult shapes grow beyond the shape of mainland adults, indicating peramorphosis. Finally, we computed shape distances (the procrustes distance between group means) for each population. The groups being compared were juveniles (individuals with 0 to 1 LAG), and adults (individuals with >1 LAG) of both populations.

### Sexual selection and dimorphism

We predicted stronger sexual selection on the island. In lizards, sexual selection is deemed to increase head width in males. This trait is correlated with male performance in male-male contests over mates, since males with wider heads have more muscle mass in the head and a higher associated bite force [[54,108-110] on *Podarcis*). We measured head widths of adults during the 2006-2008 period (Licosa Island: n = 80, Punta Licosa, n = 92). Then, we regressed log head widths against log SVL to factor out potential allometric effects, and compared residuals between the two populations (further partitioned by sex) by means of two-factor ANOVA taking sex, population, and the interaction of these two variables in the model.

Sexual size dimorphism was investigated as well by taking SVL ratio of males/females. We used SVL of individuals of known age and then compared size dimorphism between the two populations. SVL is a very good proxy for body size in lizards [111]. In addition, sexual shape dimorphism was investigated by using head width data. We repeated the tests using year of collection as a factor. No significant differences between years were found, and we therefore present an analysis of pooled data here.

## Authors' contributions

PR and DF conceived the experiments. PR and FC performed shape analyses. FMG performed LAG analyses. MT, DF, DMM, GP, and CM performed MCRs sequencing, polymorphism and quantitative assay analyses. DF, DR, GP and FC performed lab and field work. All authors participated in writing, reading and approving the manuscript.

## Supplementary Material

Additional file 1 **Supplementary materials**. The additional data file contains illustrative material, methodological details about field work, shape analysis, LAG analysis, and genetic analyses. Click here for file
