# Supplementary material for: The blue lizard spandrel and the island syndrome
Source: BMC Evol Biol. 2010 Sep 20;10:289. doi: 10.1186/1471-2148-10-289 (PMC2949876; doi:10.1186/1471-2148-10-289)
Supplement: Additional file 1 — Supplementary materials. The additional data file contains illustrative material, methodological details about field work, shape analysis, LAG analysis, and genetic analyses. [file 1471-2148-10-289-S1.DOC]

**Supplementary material**

Supplementary material is arranged as follows:

- In Figure S1 we report a sample showing the estimation of LAGs.
- The second part (figures S2-9; Tables S3,4) concerns the landmark configuration we used to model head shape and development, and the results of linear morphometrics (S5) and geometric morphometrics (S2-4, S6-9) analyses.

The third part explains how we computed population density estimates, its relationship with summer rainfall, and shows the location of our sampling transects (Table S19, Figure S10).

The fourth part reports allele polymorphism in MC1R for both populations (Tables S11).

**PART 1**

**Figure S1 –** Representative cross section of phalanx of *Podarcis sicula*. A) Insular female (SVL = 75mm). B) mainland female (SVL = 62 mm). Arrows indicate lines of arrested growth (LAGs). RL = reversal line. Both individuals were estimated to be 3 years old.

**
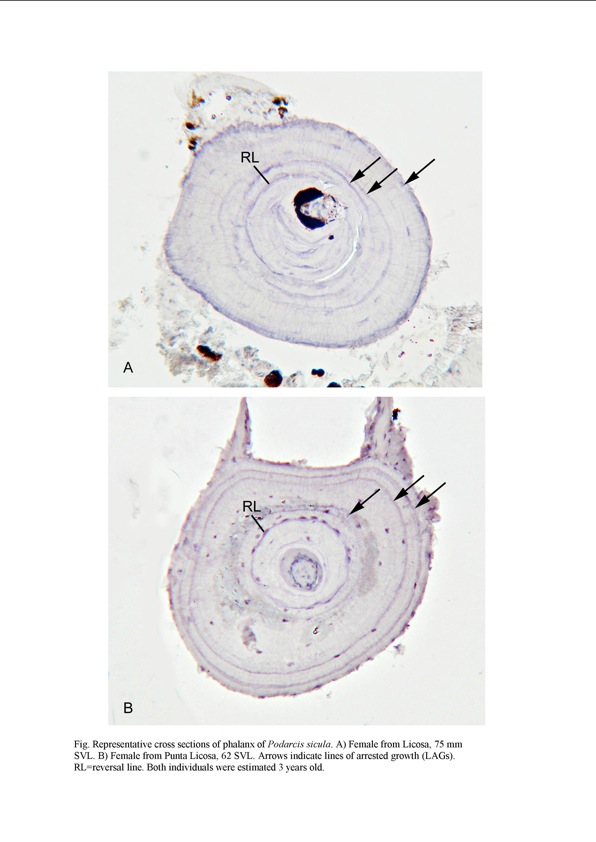
**

**PART 2**

**Geometric and linear morphometrics**

Figure S2 – The figure illustrates how landmarks were placed unilaterally on lizard heads. The landmark digitalization protocol follows the paper by Kaliontzopoulou, Carretero e Llorente (Journal of Morphology 268:152–165 (2007), for the landmarks from 2 to 11 and from 13 to 15, whereas we chose landmarks 1 and 12 on our own.


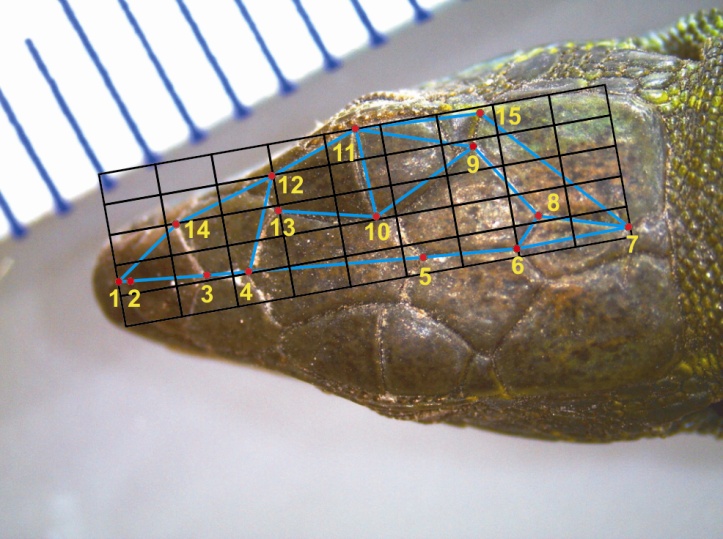


| Table S3 | Landmarks and their anatomical position |
| --- | --- |
| Landmark # | Description |
| 1 | Posterior border of the rostral |
| 2 | Anterior border of the frontonasal |
| 3 | Posterior border of the frontonasal |
| 4 | Anterior border of the frontal |
| 5 | Posterior border of the frontal |
| 6 | Anterior border of the interparietal |
| 7 | Posterior borders of the interparietal |
| 8 | Middle-anterior border of the interparietal |
| 9 | Lateral border of the frontoparietal |
| 10 | Joint between the two middle supraoculars and the frontal |
| 11 | Joint of the two middle supraoculars and the supraciliary granules |
| 12 | External joint of the anterior-middle of supraocular and the 1st supraocular |
| 13 | Middle-anterior borders of the frontal |
| 14 | Middle-posterior borders of the frontonasal |
| 15 | Posterior border of the last supraocular |

Table S4 - Distribution of head shape variance explained by each Relative Warp (RW). SV = singular value for each eigenvector. The variance explained per RW (%) and the cumulative variance (Cum %) are reported.

|  | | | |
| --- | --- | --- | --- |
| Singular values and percent explained for relative warps: | | | |
| RW | SV | % | Cum% |
| 1 | 0.29801 | 25.10% | 25.10% |
| 2 | 0.18218 | 9.38% | 34.48% |
| 3 | 0.17861 | 9.01% | 43.49% |
| 4 | 0.17525 | 8.68% | 52.17% |
| 5 | 0.16774 | 7.95% | 60.13% |
| 6 | 0.15073 | 6.42% | 66.55% |
| 7 | 0.14217 | 5.71% | 72.26% |
| 8 | 0.13196 | 4.92% | 77.18% |
| 9 | 0.11047 | 3.45% | 80.63% |
| 10 | 0.10071 | 2.87% | 83.50% |
| 11 | 0.09639 | 2.63% | 86.12% |
| 12 | 0.08588 | 2.08% | 88.21% |
| 13 | 0.08334 | 1.96% | 90.17% |
| 14 | 0.07542 | 1.61% | 91.78% |
| 15 | 0.07410 | 1.55% | 93.33% |
| 16 | 0.06667 | 1.26% | 94.58% |

Figure S5 – Head width allometry for the two populations (in mm, linear measurements).


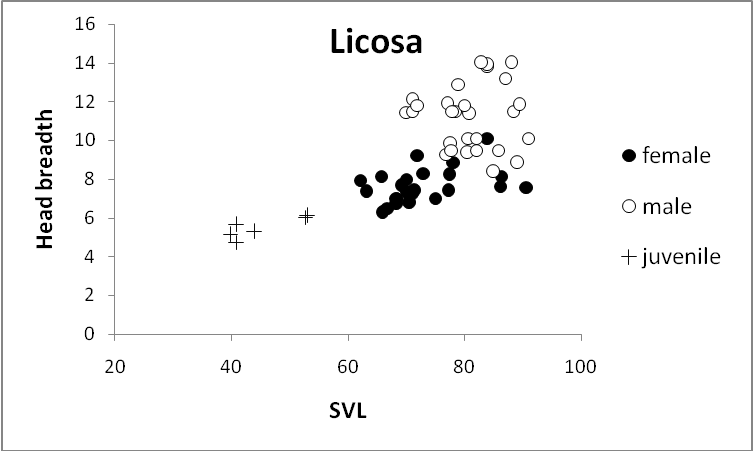

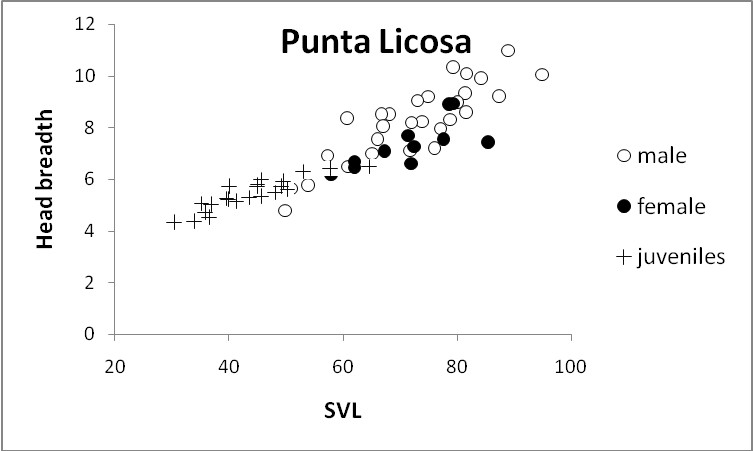


Figure S6 - Below we report an ideal representation of the ontogenetic trajectory of head shape development for the two populations analyzed; Licosa (blue line) and Punta Licosa (green line). The Licosa lizards’ ontogeny has statistically the same slope, and points in the same direction of the mainland individuals’, although its range values are shifted to peramorphic shapes both in juveniles and adults, by means of developmental changes confined in early morphogenesis.

Peramorphosis

Paedomorphosis

Mainland lizards ontogeny

Insular lizards ontogeny

AGE

HEAD SHAPE

Figure S7 - The two plots below show the distribution of individuals per population and sex. Axes represent orthogonal vectors of maximum variation, “Relative Warps” (RWs) in the geometric morphometrics nomenclature; x-axis = RW1, y-axis = RW2:


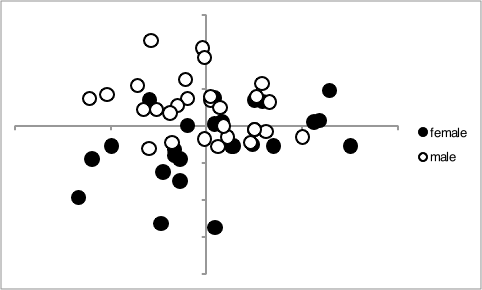


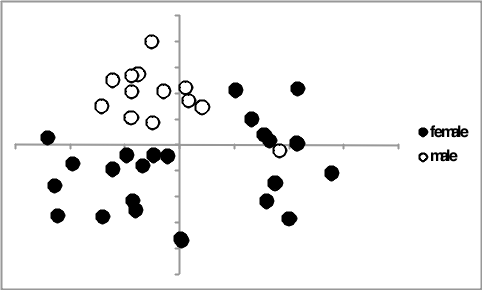


Punta Licosa

Licosa

Figure S8 - Shape deformation associated to the plots in figure S7. Consensus (= average) shape for sex and for population are shown. The analyses were performed with the software Morphologika^2^ v2.5, by O’Higgins and Jones (2006), available at <http://hyms.fme.googlepages.com/resources> .


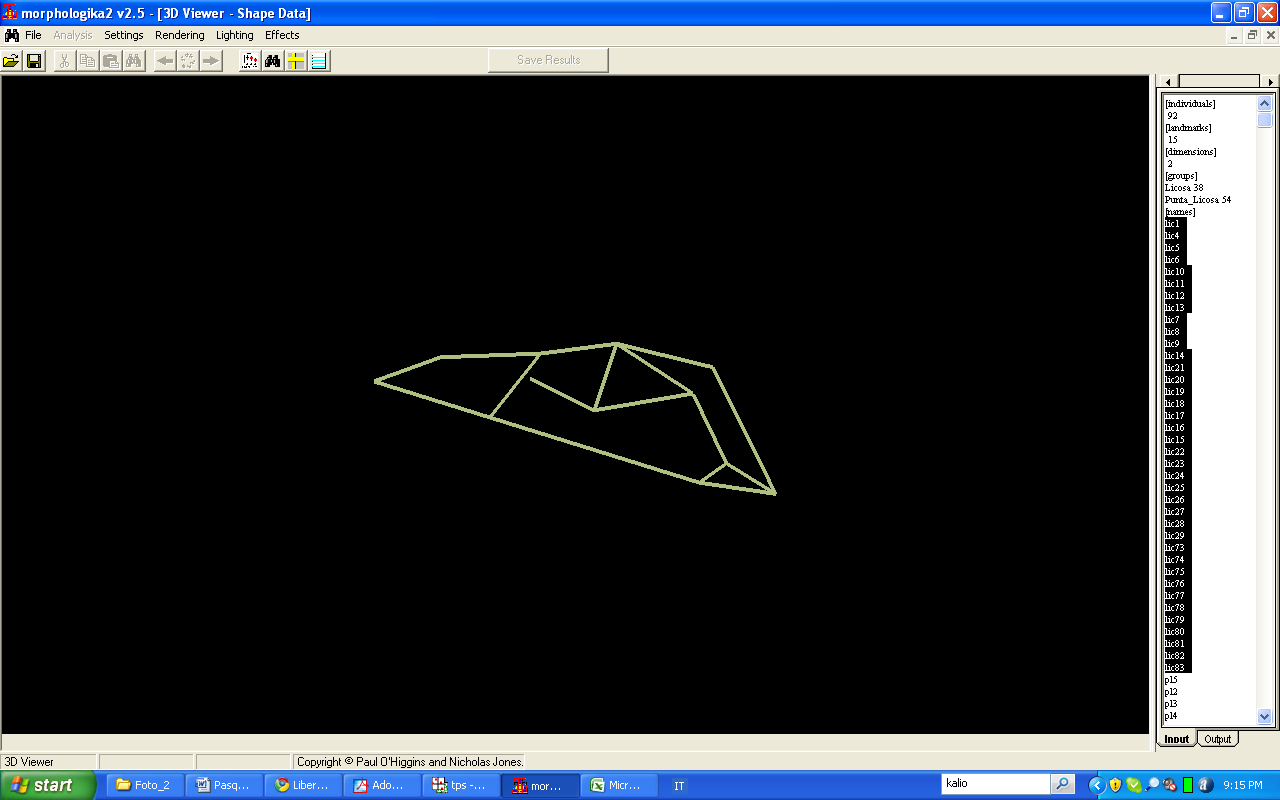

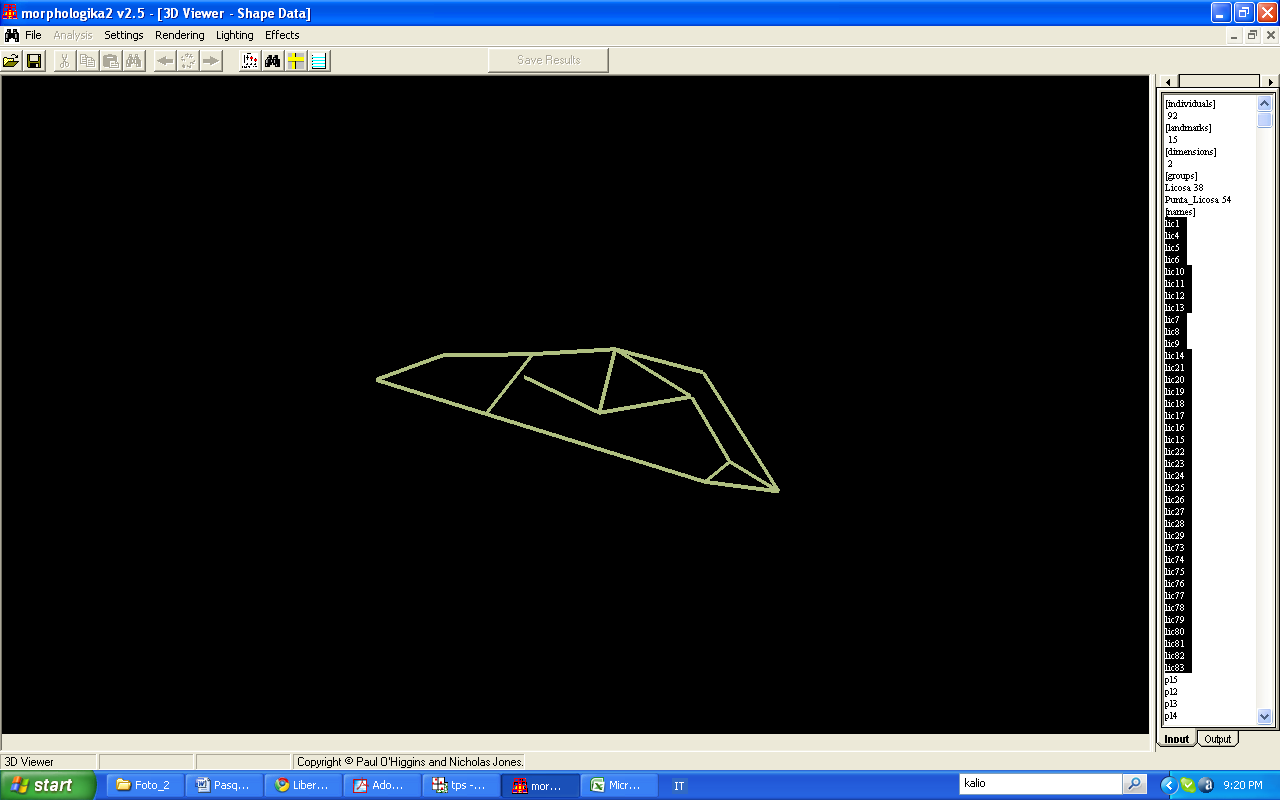


Punta Licosa


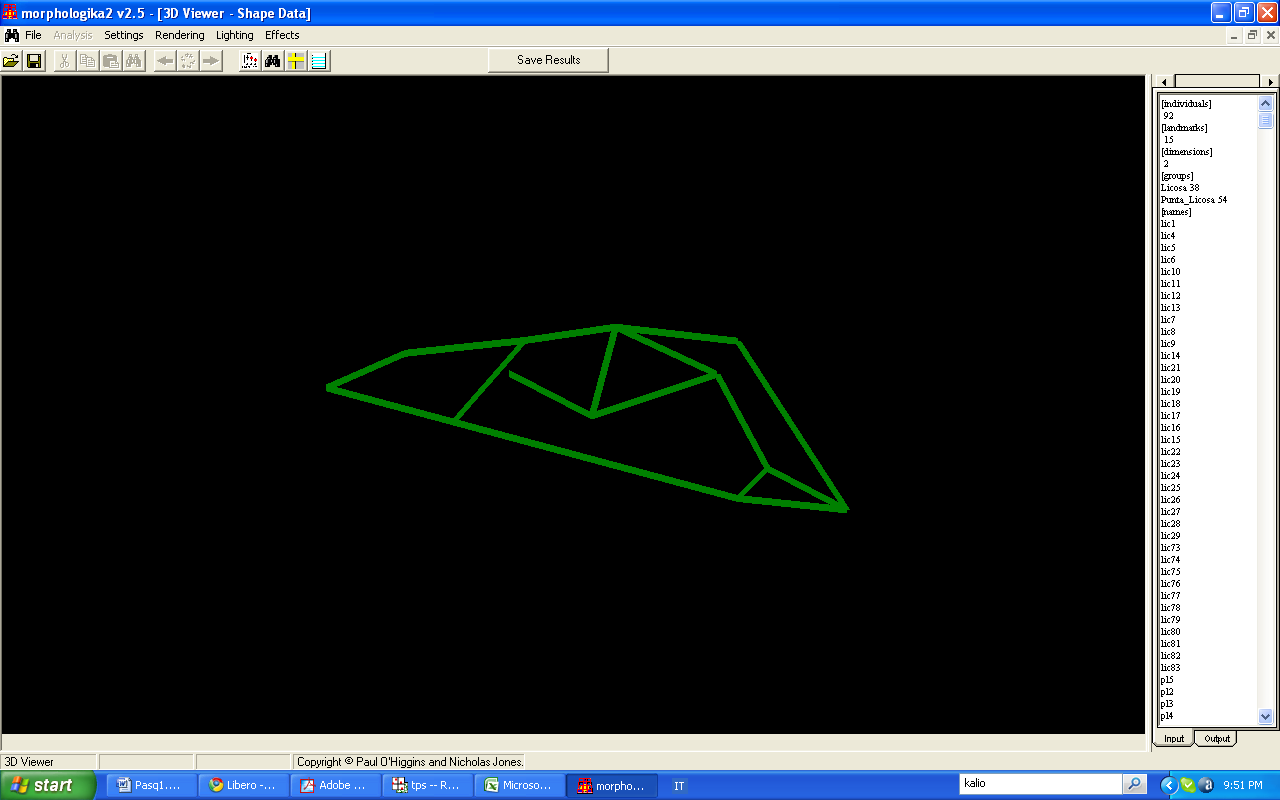

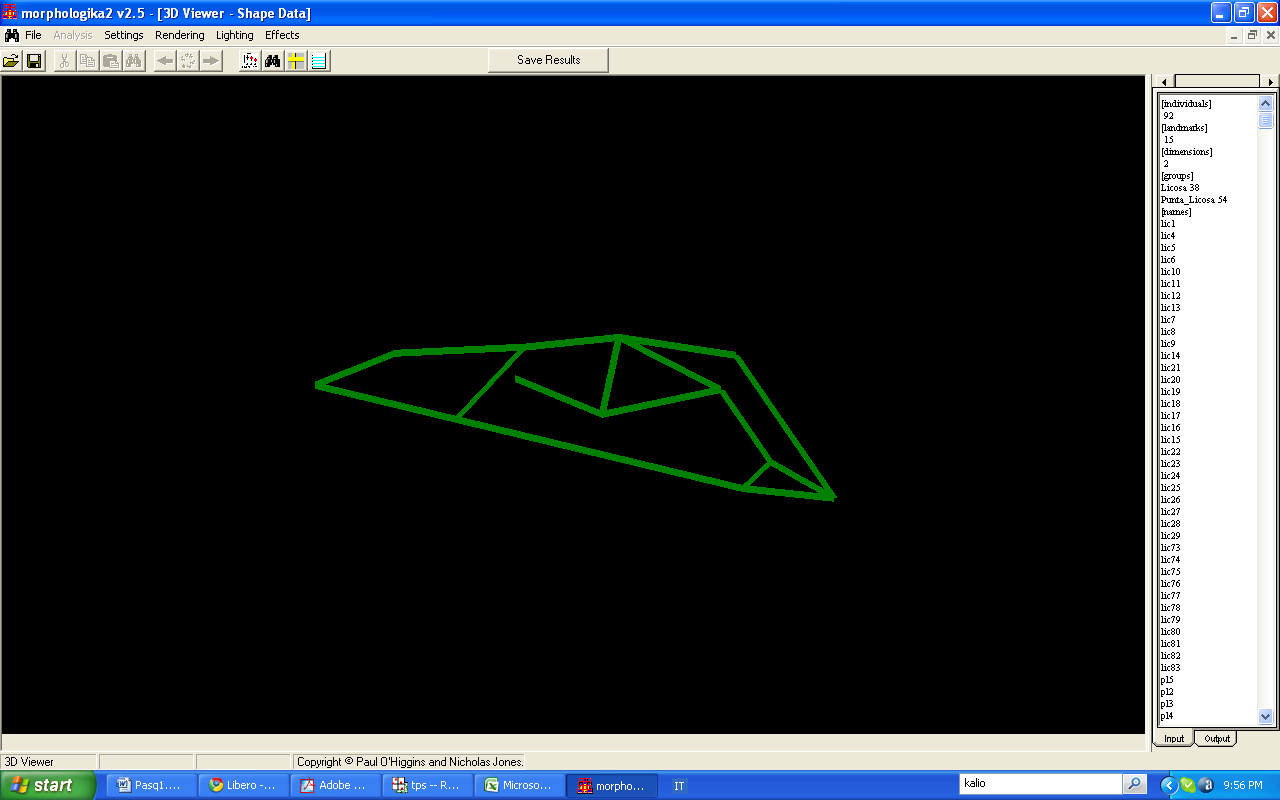


Licosa (insular)

Male

Male

Female

Female

Figure S9- RW1 / RW2 scatterplot of the two populations. Size (SVL) change occurs along RW1. It is evident that young individuals in Licosa appear “adultized” in comparison to Punta Licosa (mainland) juveniles. Individual with a number of LAGs between 0 and 1 were considered to be “juveniles”.


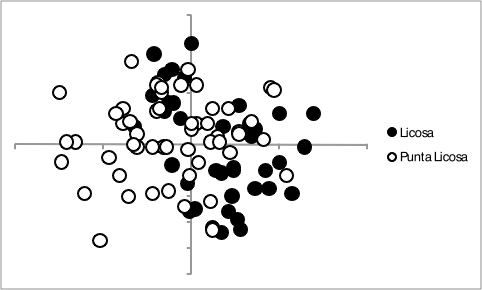


**SVL**

**PART 3**

**Population density estimates**

Population size was estimated by means of distance sampling. In the mainland and on the island we divided ecologically similar spatial areas in 5 transect lines (Fig. S10) and walked along transects once per day for 5 consecutive days between h9:00 and h11:00. The total length of transects is the same for the two populations. When a lizard was sighted, its exact position was recorded by painted markers on the spot. All sightings were made from the line. When all transects had been surveyed, the lines were re-walked and exact locations of the lizards were measured to the line. Perpendicular distances were measured from the closest marker. Abundance estimated from transect data was calculated using Program DISTANCE (Thomas et al, see DOI: 10.1111/j.1365-2664.2009.01737.x). DISTANCE fits the probability density function to model the decrease in detection of objects at greater distances from the transect line. A truncation distance of 5m was used in all analyses. Data were analyzed over 5 consecutive years.

Figure S10 - A) Study area from a satellite view
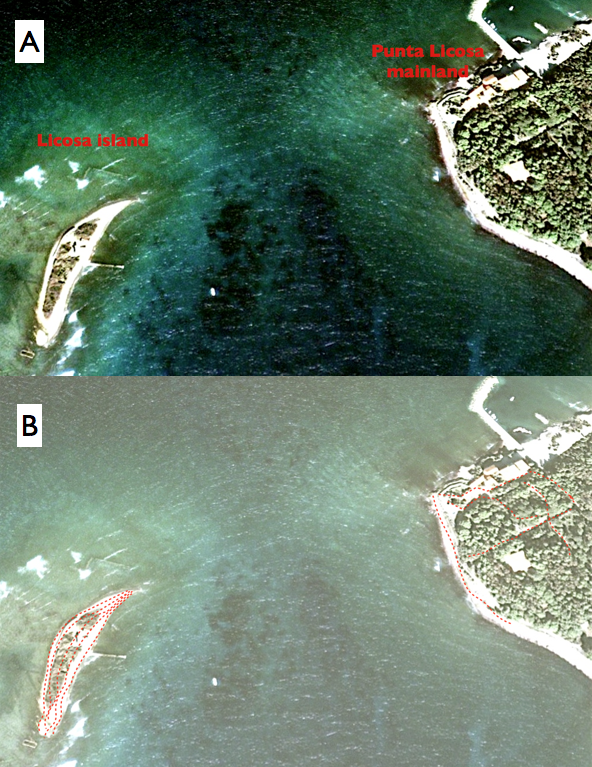
(http://earth.google.com/intl/it/licensepro.html); B) Spatial location of transects are shown (dotted red lines).

Population density was strongly fluctuating on the Island and roughly constant on the mainland during our 5 years-long survey. Density on Licosa in one year is significantly correlated with total rainfall in the previous year. This might be explained by the absence of freshwater sources on Licosa. No significant correlation was detected for the mainland population.

**Table S11 – Total rainfall from April to August for the 2003-2007 period at the meteorological station closest to the study area (Policastro Bussentino station,** [**http://www.sito.regione.campania.it/agricoltura/meteo/agrometeo.htm**](http://www.sito.regione.campania.it/agricoltura/meteo/agrometeo.htm)**) and population density statistics for both populations over the 2004-2008 period. Correlation test statistics are reported in the bottom rows.**

|  |  |  | Population Density estimates | | |
| --- | --- | --- | --- | --- | --- |
| Total rainfall (in mm) over April to August | |  | Year | Licosa | Punta Licosa |
| 2003 | 59.5 |  | 2004 | 148.1 | 355.3 |
| 2004 | 246.8 |  | 2005 | 246.2 | 341.1 |
| 2005 | 157.1 |  | 2006 | 231.2 | 360.9 |
| 2006 | 319.3 |  | 2007 | 241.7 | 388 |
| 2007 | 96.7 |  | 2008 | 159.4 | 350.9 |
|  |  |  |  |  |  |
| Correlation test statistics | | |  |  |  |
| Person Product Moment | | |  | 0.881 | 0.369 |
| t |  |  |  | 3.225 | 1.055 |
| p |  |  |  | 0.048 | 0.52 |

**PART 4**

**Table S12 - Polymorphism of MC1R sequences**

We surveyed molecular variation in Lizard MC1R from the island and the mainland lizards, and identified several MC1R polymorphisms. Below we report nucleotide change position and distribution per population (Lic = Licosa; PLic = Punta Licosa):

| **Nucleotide**  **change*** | **Protein**  **change** | **Total # of Lizards showing the variation** | **# of Lizards showing the variation per population** |
| --- | --- | --- | --- |
| C.329C>T | p.Ile110Thr | 16/40 | 11Lic-5PLic |
| C.351C>T | No | 10/40 | 0Lic-10Plic |
| C.944G>A | No | 24/40 | 8Lic-16Plic |

For nucleotide numbering, the first A of the initiator ATG codon is nucleotide +1 of the MC1R sequence (GenBank Accession number: GU225767.1).
